# DockRMSD: an open-source tool for atom mapping and RMSD calculation of symmetric molecules through graph isomorphism

**DOI:** 10.1186/s13321-019-0362-7

**Published:** 2019-06-07

**Authors:** Eric W. Bell, Yang Zhang

**Affiliations:** Department of Computational Medicine and Bioinformatics, University of Michigan, 100 Washtenaw Avenue, Ann Arbor, MI 48109-2218 USA

**Keywords:** Symmetric molecules, Protein–ligand docking, Ligand pose comparison

## Abstract

Comparison of ligand poses generated by protein–ligand docking programs has often been carried out with the assumption of direct atomic correspondence between ligand structures. However, this correspondence is not necessarily chemically relevant for symmetric molecules and can lead to an artificial inflation of ligand pose distance metrics, particularly those that depend on receptor superposition (rather than ligand superposition), such as docking root mean square deviation (RMSD). Several of the commonly-used RMSD calculation algorithms that correct for molecular symmetry do not take into account the bonding structure of molecules and can therefore result in non-physical atomic mapping. Here, we present DockRMSD, a docking pose distance calculator that converts the symmetry correction to a graph isomorphism searching problem, in which the optimal atomic mapping and RMSD calculation are performed by an exhaustive and fast matching search of all isomorphisms of the ligand structure graph. We show through evaluation of docking poses generated by AutoDock Vina on the CSAR Hi-Q set that DockRMSD is capable of deterministically identifying the minimum symmetry-corrected RMSD and is able to do so without significant loss of computational efficiency compared to other methods. The open-source DockRMSD program can be conveniently integrated with various docking pipelines to assist with accurate atomic mapping and RMSD calculations, which can therefore help improve docking performance, especially for ligand molecules with complicated structural symmetry.

## Introduction

Computer-aided drug design, in particular protein–ligand docking, has brought about the discovery of many biologically active drugs [1, 2]. In many protein–ligand docking programs, a flexible small molecule structure is docked in a rigid protein receptor structure in order to find the optimal binding conformation and affinity of the small molecule within the protein binding pocket. Since the ability of these programs to accurately assess binding affinity is dependent on their ability to find the optimal conformation of the ligand in the protein binding pocket, docking programs are often benchmarked by their ability to reproduce the native binding pose of a ligand from a protein–ligand complex crystal structure. A common metric used to evaluate distance between the predicted pose and the native pose, given a superposition of their protein receptor structures, is the root mean square deviation (RMSD) between their respective atoms (Eq. 1):

1 $$RMSD = \sqrt {\frac{1}{N}\mathop \sum \limits_{i = 1}^{N} d_{i}^{2} }$$

where *N* is the number of atoms in the ligand, and *d*_*i*_ is the Euclidean distance between the *i*th pair of *corresponding* atoms.

**Fig. 1:**
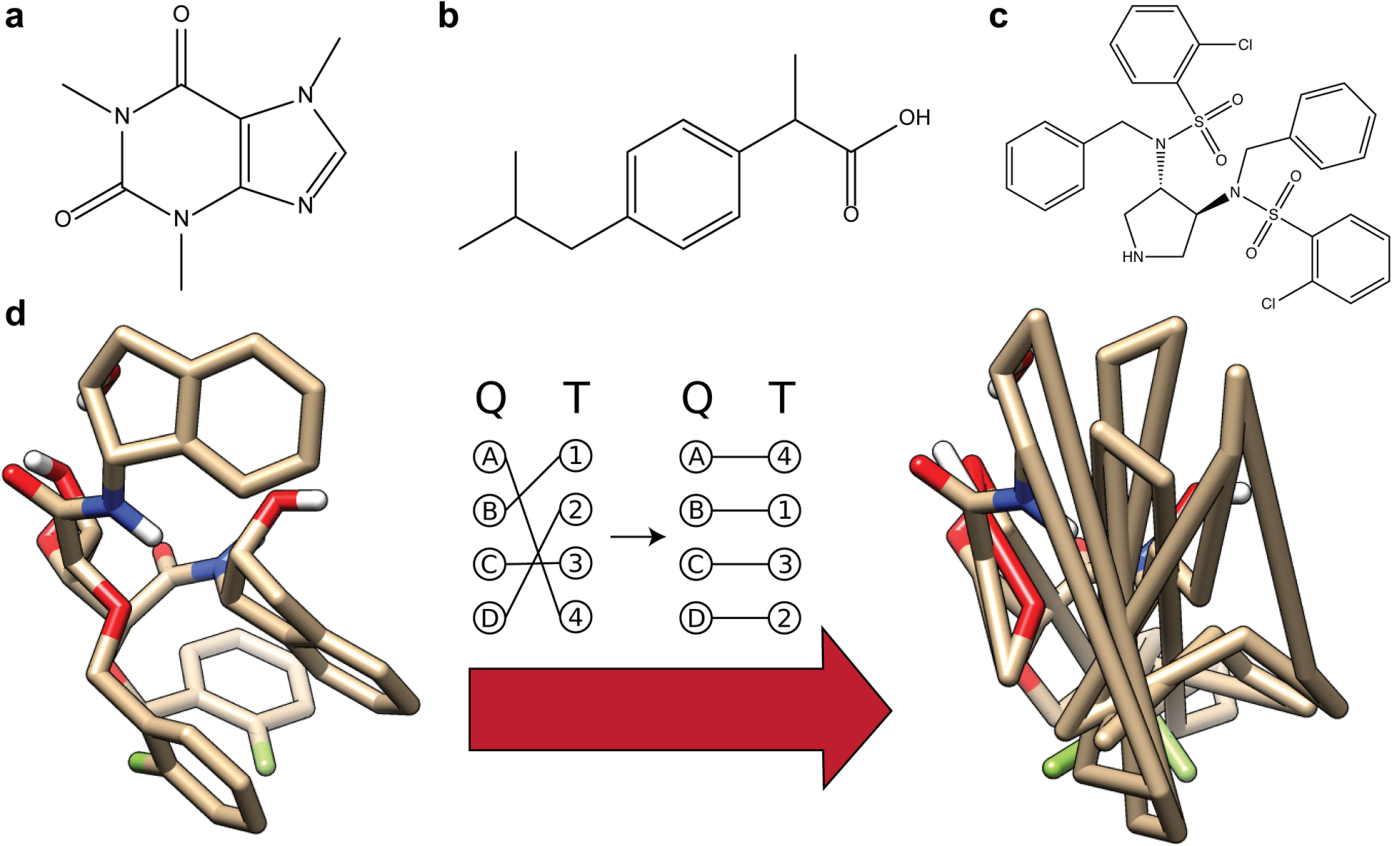
Examples of **a** an asymmetric ligand (PDB Ligand ID: CFF); **b** a slightly symmetric ligand (PDB Ligand ID: IBP); **c** a highly symmetric ligand (PDB Ligand ID: QN3). **d** An example ligand structure (left) and the resulting ligand structure when the atoms are reordered according to the optimal query-template atomic correspondence generated by the Hungarian method (right). Since the Hungarian method only takes atom type into account and not the bonds between atoms, the hypothetical molecule proposed by the Hungarian correspondence is physically impossible

Docking RMSD can be most naïvely calculated with the assumption of direct atomic correspondence, or in other words, the assumption that the atomic labels between ligand structures in the given structure files are ordered and should remain static in the docking process. This assumption holds for asymmetric molecules like caffeine (Fig. 1a), but this correspondence is not always practically relevant for molecules with symmetric functional groups (e.g. ibuprofen, Fig. 1b) or whole-molecule symmetry (e.g. the pyrrolidine-based inhibitor of HIV-1 protease [3] in Fig. 1c), as they can give rise to binding poses that are identical in terms of chemistry, but not in terms of correspondence. Here, ibuprofen and HIV-1 protease pyrrolidine-based inhibitor have been chosen as illustrative examples, although there are various other molecules with symmetric structures in which naïve correspondence can result in false inflation of RMSD (e.g. the inhibitor BEA403 [4], c-di-GMP [5], etc.). For example, if one were to perfectly overlap two benzene molecules, their docking RMSD would have a value of zero. If one were to then rotate one molecule along one of its axes of symmetry until the two structures overlapped perfectly again, their docking RMSD should be zero due to the chemical identity of the overlap; since the overlapping atoms are differently labeled between the two molecules in this example, naïve docking RMSD would have a nonzero value. Therefore, molecular symmetry needs to be taken into account in order to derive an accurate docking RMSD value.

Several docking programs have implemented docking RMSD modules to accommodate ligand symmetry. AutoDock Vina [6] was one of the first to implement symmetry correction in docking RMSD calculation, providing a module that creates correspondence by mapping each atom of one pose to the closest atom of the same type from the other pose. However, this method allows the potential for atoms that are close between the two structures to be used repeatedly and atoms that are distant to not be used at all. In response to this, Allen and Rizzo [7] implemented their own docking RMSD calculator in DOCK6 [8] which presents atomic correspondence mapping as a cost-minimization assignment problem, solved by using the Hungarian algorithm [9, 10]. However, considering the mapping problem in this way ignores the bonding structure of the ligand, and can potentially provide nonphysical assignments (Fig. 1d) and docking RMSD values that are lower than what should be physically possible. Several other docking programs, such as GOLD [11], AmberTools [12], and Glide [13, 14] also contain modules that calculate symmetry-corrected RMSD, but these modules generally do not publicly offer thoroughly detailed explanations of their symmetry correction algorithms and demand that the user install a much larger package to calculate symmetry corrected RMSD. Finally, OpenBabel [15] contains a C++ open source tool, *obrms*, that considers symmetry correction as a graph isomorphism problem, solved by the VF2 algorithm [16], but also currently requires that the user install the entirety of OpenBabel to use this tool. Docking RMSD calculated by these modules is distinct from conformational distance metrics calculated by programs such as LS-align [17] and RDKit [18], as these metrics are based on a superposition of the ligand structures themselves, not the receptor on which they are docked. Such a superposition is inappropriate for evaluation of docking poses due to the lack of consideration of the position and orientation of the ligand relative to the receptor; it is more appropriate for purely cheminformatic problems, such as ligand structural similarity comparisons. Therefore, there exists a need for a universal docking RMSD calculation module that properly considers molecular symmetry and does so with a clear, detailed description of its methodology.

Here we propose a new, open-source module, DockRMSD, to solve the atom mapping issue for symmetric molecular structures through graph isomorphism, where the optimal docking RMSD is calculated by searching through a pruned state space of all isomorphic mappings between two molecular structures. Source code in C, compiled binaries, and a web server implementation of DockRMSD are made freely available at the DockRMSD web site [19].

## Implementation

**Fig. 2:**
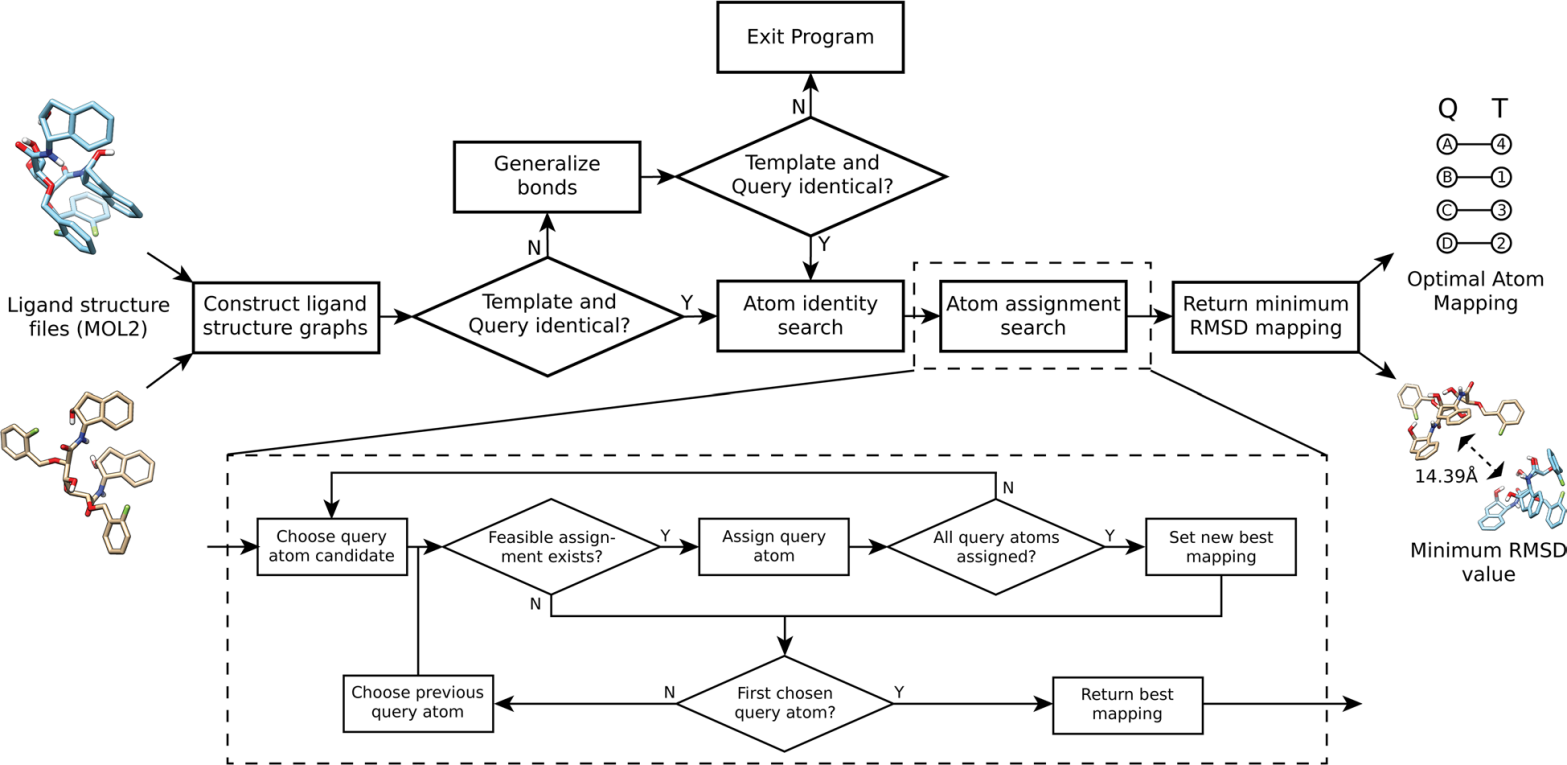
The DockRMSD algorithm. DockRMSD calculates the optimal atom mapping and RMSD value for any given pair of poses for the same ligand, input as a pair of MOL2 structure files

A general overview of the DockRMSD algorithm is presented in Fig. 2. To begin, the user provides a pair of structure files in MOL2 format, each containing a specific pose of the same ligand. The first file is arbitrarily defined as the “query” structure and the second as the “template” structure, for convenience of description. The elements of the heavy (non-H) atoms present in each structure, the coordinates of those atoms, and the bonding network between the pairs of atoms are read from the structure files. Subsequently, the atom and bond sets are compared in order to ensure that the two structures are of the same ligand molecule. Bonds are represented by a symmetric two-dimensional array which contains a string corresponding to bond type (single = “1”, double = “2”, aromatic = “ar”, etc.) between bonded atoms *i* and *j* at array position [*i*, *j*], and contains empty strings otherwise. If the bond types do not agree between the two files, the bond network is stripped of bond types, preserving only which atoms are bonded.

Once the ligand structural information has been extracted, the next step is to determine the set of template atoms to which each query atom is chemically identical, referred to as the atom identity search. For each atom of the query structure, all atoms of the template structure of the same element are initially considered to be candidate mapping partners. Then, the set of atoms that the query atom is bonded to, as well as the bond types between them, is evaluated against the set of atoms and bonds present for each candidate mapping partner in the template; candidate template atoms are eliminated if their bonding structure does not match the query atom. This process is repeated, checking for identity between not only their set of bonded neighbor atoms, but the neighbors of those neighbor atoms as well, once again removing candidate atoms if the sets are not identical. A deeper search involving further neighbor atoms was attempted, but it was found that including more neighbors ultimately did not change the final optimal correspondence. Therefore, the identity search stops at this neighbor atom depth in order to minimize unnecessary neighbor set comparisons and optimize runtime. If more than one candidate remains, there is likely more than one atom in the template that is chemically identical to the query atom, meaning that the ligand has some degree of symmetry. Once the atom identity search is complete, each query atom will have a set of template atoms that are chemically equivalent to that query atom. For a completely asymmetric molecule (Fig. 1a), each query atom will only have one corresponding template atom. Therefore, calculating the optimal RMSD for these asymmetric molecules is a simple task of matching each query atom to its respective template atom and returning the RMSD calculated from this correspondence. However, for symmetric molecules, one must search through all possible assignments of template atoms in order to find the mapping whose RMSD is minimal. The putative computational expense of this search is calculated as the total number of possible mappings, i.e. the product of each query atoms’ candidate atom set length.

In order to find the deterministically optimal mapping between query and template atoms, an exhaustive assignment search reminiscent of the VF2 algorithm [16] coupled with Dead-End Elimination (DEE) [20] is implemented. In this procedure, query atoms are iteratively assigned the template label that provides the smallest squared interpose distance and can feasibly added to the existing assignments. The first of these feasibility criteria is if the candidate template atom that is being assigned has already been assigned to a previous query atom, this assignment is disallowed. This restriction ensures that all mappings are one-to-one such that no template atom is mapped to more than one query atom. Second, if the query atom that is currently being mapped is bonded to already mapped atoms, the template bonding network is checked to ensure that a bond also exists in the template between the labels given to those atoms from the query. If the bonds being formed by query atom assignment are not supposed to be formed according to the template, the proposed assignment is not feasible. Finally, the last feasibility criterion is DEE, which ceases assignment of a particular atom if all subsequent feasible assignments would result in an RMSD larger than the smallest heretofore observed RMSD (which is infinity if no RMSD has yet been observed). The query atoms are mapped in order of number of possible template labels (smallest first), then number of bonds to already mapped query atoms (largest first), and finally, the order in which they appear in the query file (smallest first). Once all query atoms have been assigned to template atoms, this correspondence is used to calculate RMSD. The minimum RMSD for all mappings and the mapping that gave rise to that RMSD are then printed by the program. In addition, the number of possible mappings is printed.

## Results and discussion

### Docking conformation dataset and generation

To evaluate DockRMSD’s symmetry correction and the reliability of the greedy search heuristic, we generated docking conformations based on the CSAR Hi-Q protein-ligand dataset [21]. This dataset contains 343 protein structures with manually refined binding pockets, each in complex with their respective ligand, where the docking decoy conformations have been generated by ourselves using the AutoDock Vina program [6]. For each protein–ligand pair, the native ligand structure was removed, conformationally randomized using OpenBabel [15], and re-docked into the binding pocket using AutoDock Vina [6]. The generation of input PDBQT files for docking and the output file conversion from PDBQT to MOL2 was performed by OpenBabel. Docking RMSD was calculated between all 10 possible pairwise combinations of the top five poses generated from a single re-docking experiment, leading to a total of 3430 RMSD calculations (10 per protein–ligand pair, 343 protein–ligand pairs in total). All 3430 calculations were performed using a list of different programs on a Red Hat Enterprise Linux machine with an Intel i5-4590 CPU @ 3.30 GHz. The average total walltime for all 3430 RMSD calculations was 4.8 ± 0.7 s, 5.3 ± 0.9 s, and 60.1 ± 0.1 s for DockRMSD, naïve RMSD, and *obrms*, respectively (see “DockRMSD runtime comparison” section for a more detailed runtime analysis).

Here, naïve RMSD calculations relative to the native crystal structure pose were not calculated because the AutoDock Vina ligand preparation process removes direct atomic correspondence between the redocked ligand and the native ligand. AutoDock Vina re-orders the atoms of the ligand according to the ligand’s torsional tree, and therefore, all Vina poses have direct correspondence with each other, but not with the original native ligand structure. Therefore, only programs that can find atomic correspondence between files can be used to compare the Vina poses to the native crystal structure pose. This limitation is why the dataset used to evaluate the programs consists only of docked poses; direct correspondence cannot be drawn between the native crystal structure and Vina-generated poses. Ligand structures have been visualized using UCSF Chimera [22].

### Docking RMSD calculation through DockRMSD

**Fig. 3:**
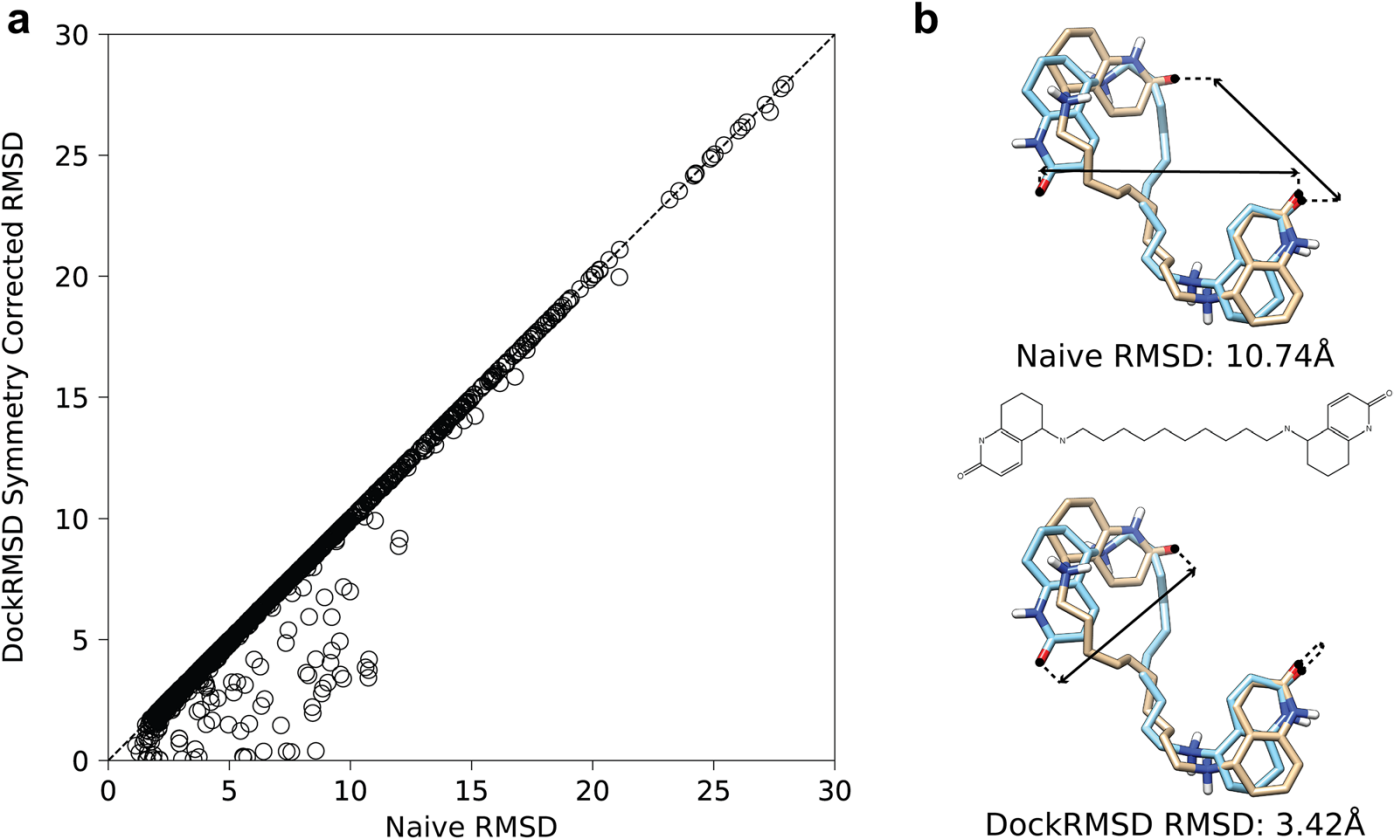
**a** Ligand RMSD calculated by DockRMSD versus that by the naïve RMSD calculations on 1321 ligand molecules with symmetric structures. **b** An example pair of poses where naïve RMSD calculation failed to provide the optimal RMSD due to molecular symmetry (Ligand PDB ID: E10; Receptor PDB ID: 1H22 [23]). Interpose correspondence between oxygen atoms is drawn to represent the source of the RMSD disagreement by different methods

To examine the impact of symmetry correction in docking RMSD calculation, we compare in Fig. 3a the symmetry-corrected RMSD calculated from DockRMSD and the naïve RMSD which was calculated from the default atom order of the structure files. While 2109 of the 3430 cases require no symmetry correction, the remaining 1321 (38.5%) are cases where adhering to naïve RMSD artificially inflates the docking RMSD, by more than 2 Å in 54 of these cases (Table 1). The most extreme examples of this are when a ligand molecule is large and possesses a mirror plane of symmetry, and when the ligand poses roughly overlap. For these cases, determining the optimal mapping is essential because misplaced correspondence will give rise to unreasonably large interatom distances, especially when compared to the relatively small “true” RMSD. An example of a Huperzine A-based ligand of acetylcholinesterase [23] is shown in Fig. 3b, where the two halves of the molecule are chemically identical to another and by eye should have a relatively small RMSD value. DockRMSD’s calculation aligns with this rough assessment, calculating an RMSD value of 3.42 Å. However, due to the fact that the query is flipped relative to the template, naïve RMSD considers this reorientation an important distinction, and therefore calculates the RMSD to be 10.74 Å.

**Table 1:** Counts of 3430 total RMSD calculations whose error relative to the deterministic DockRMSD calculation is zero, small (nonzero but smaller than 2.0 Å), or large (greater than 2.0 Å)

	N (Error = 0)	N (0 < Error ≤ 2.0 Å)	N (Error > 2.0 Å)
Naïve	2109 (61.5%)	1267 (36.9%)	54 (1.6%)
Hungarian	161 (4.7%)	2548 (74.3%)	721 (21.0%)

**Fig. 4:**
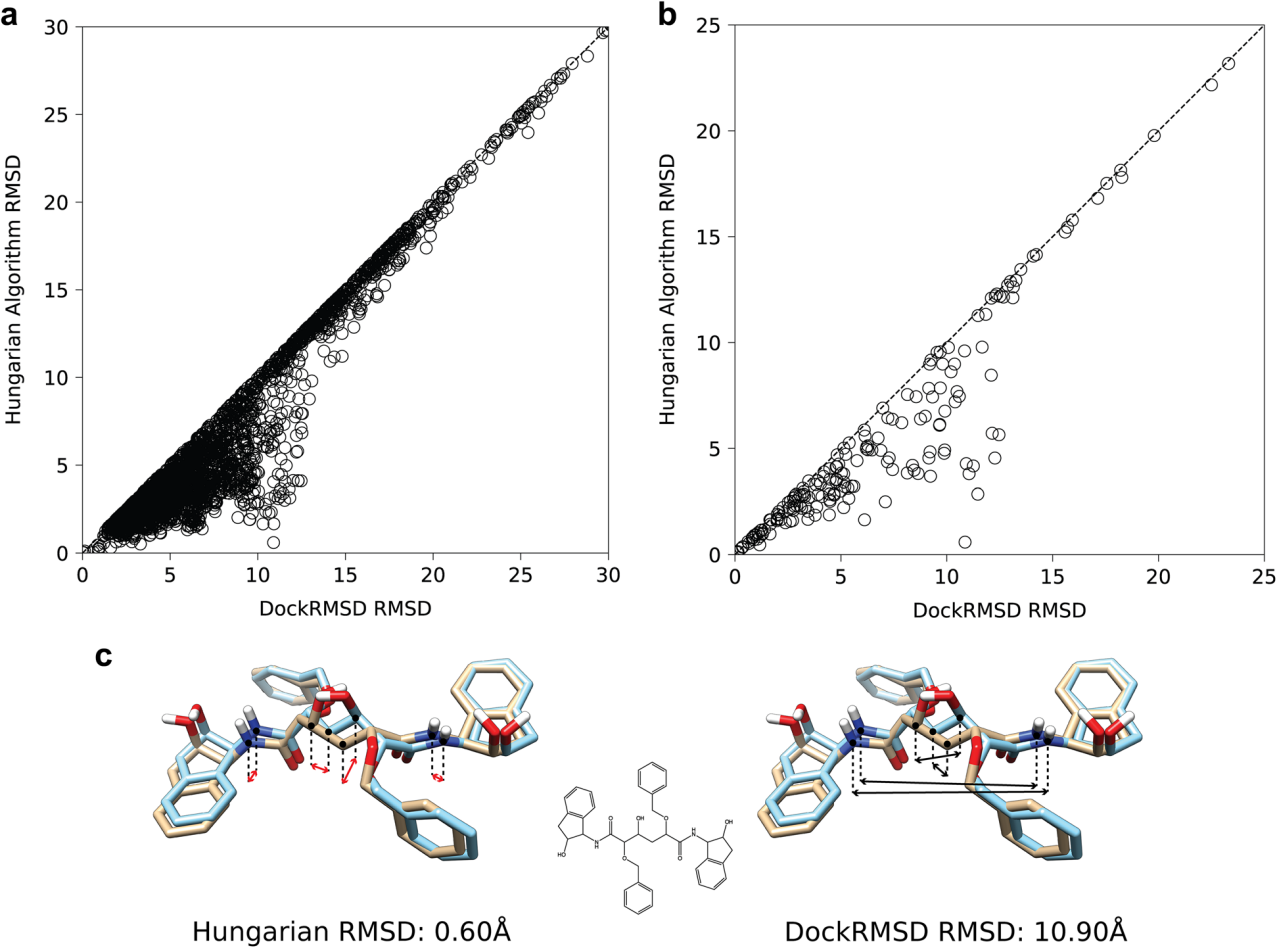
**a** Comparison of the Hungarian algorithm against DockRMSD for the 3112 molecules whose RMSD was underestimated by the Hungarian algorithm. **b** Comparison of the Hungarian algorithm against DockRMSD for the 190 molecules whose RMSD was underestimated by the Hungarian algorithm in the native ligand pose benchmark. **c** An example pair of poses where the Hungarian algorithm grossly overcorrected for symmetry due to its insensitivity to global molecular topology (Ligand PDB ID: BEG; Receptor PDB ID: 1D4I [25]). Interpose correspondence between central carbon atoms and nitrogen atoms is drawn to represent the source of the RMSD disagreement. Hungarian correspondence is drawn in red to demonstrate that the correspondence should not be allowed according to the chemical inequivalence of the atoms bonded to each atom of the pair

In Fig. 4a, we present a comparison between the RMSD of DockRMSD and that calculated by the Hungarian algorithm, which has been adopted by several established methods, such as DOCK6 [8]. In the Hungarian algorithm, the mapping is generated through iterative manipulation of a cost matrix (i.e. an interatom distance matrix) such that a pattern of zero values corresponding to the optimal assignment appears. The performance of the Hungarian algorithm was evaluated using a Python implementation of the docking RMSD calculation procedure similar to what is described by Allen and Rizzo [7]. The script uses the Python Munkres package [24] to generate query-template atomic correspondence such that assignments can only be made between atoms of the same element. As explained above, the laxness of this algorithm causes it to over-optimize and generate RMSD values below what should be possible. As a result, in nearly every case analyzed, the Hungarian algorithm generated an RMSD value below the optimal answer found by DockRMSD (3269 of 3430 RMSD calculations, 95.3%; Table 1). This implies that the over-correction issue introduced by the Hungarian algorithm is not trivial.

In contrast to the comparison between DockRMSD and naïve RMSD, the largest discrepancies between DockRMSD and the Hungarian algorithm are present in near mirror-symmetric molecules whose poses overlap almost exactly. As an illustrative example, we present in Fig. 4c a result from the HIV-1 protease inhibitor BEA425 [25], where the poses presented look nearly identical by eye, and thus, one would anticipate the RMSD value should be low. However, this molecule is not truly symmetric due to a hydroxyl group near the center of the molecule, and therefore, the two poses are not truly chemically identical. Since the Hungarian algorithm only takes into account individual atom types and not global chemical identity, cases like these fool the algorithm into accepting regions of local correspondence at the cost of properly considering which atoms are bonded. Although the algorithm generates lower RMSD values, these values do not reflect correct correspondence of the atomic mapping derived from the ligand bonding structures.

Here, it is noted that the above RMSD calculations are performed on the AutoDock Vina docked conformations, which was chosen purely to enable the comparison of different RMSD calculation programs with direct correspondence. In fact, one of the most common applications of ligand RMSD calculation is for benchmarking experiments that evaluate a docking program’s ability to produce ligand poses that closely resemble the native conformation. In such experiments, poses are typically considered “near-native” if their RMSD relative to the native pose is  ≤  2.0 Å. In order to examine the performance of different programs with respect to this task, the top ranked AutoDock Vina pose for each of the 343 protein–ligand pairs was compared against the crystal structure pose of the ligand as provided by the CSAR Hi-Q set using both DockRMSD and the Hungarian algorithm, the results of which are presented in Fig. 4b and Table 2. It was shown that in 190 of the 343 cases, the Hungarian algorithm resulted in a lower value than the optimal value as determined by DockRMSD, where 10 of them would have resulted in a false positive classification of a “near native” pose. These results demonstrate that evaluation of a docking algorithm by RMSD values using incorrect atomic correspondences can lead to artificial inflations of docking results.

**Table 2:** A contingency table for 343 RMSD calculations between docked ligand poses and their respective native crystal structure ligand poses, calculated both by DockRMSD and the Hungarian algorithm

	Hungarian RMSD > 2.0 Å	Hungarian RMSD ≤ 2.0 Å
DockRMSD > 2.0 Å	157	10
DockRMSD ≤ 2.0 Å	0	176

### DockRMSD runtime comparison

**Fig. 5:**
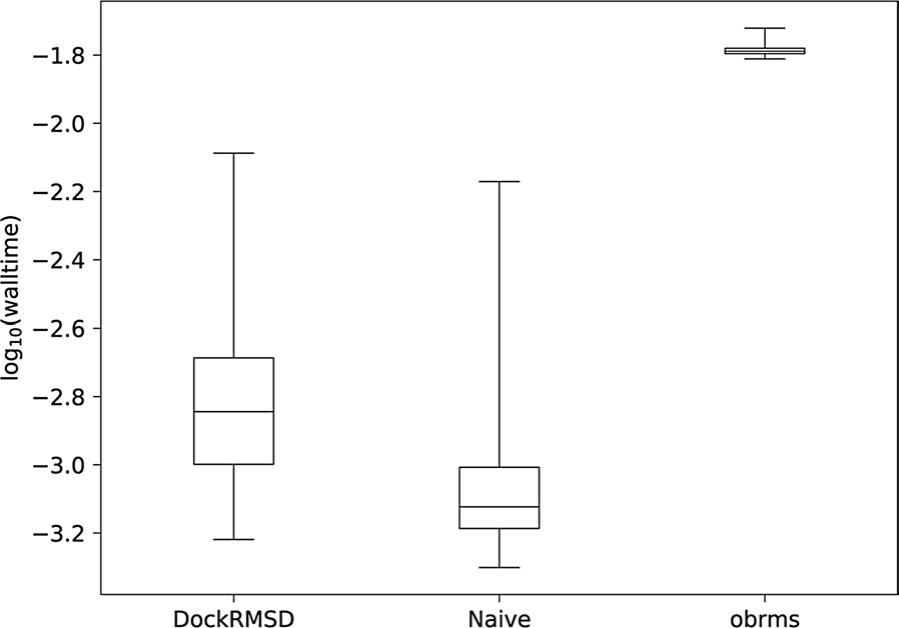
Box and whisker plots of the walltime distributions (in log_10_(sec)) for each of the 3430 RMSD calculations as calculated by symmetry-corrected DockRMSD, symmetry-uncorrected naïve RMSD, and symmetry-corrected *obrms*

In order to evaluate the runtime efficiency of DockRMSD, both naïve and symmetry-corrected RMSD calculations on all 3430 pose pairs were compared to the runtimes of *obrms*. The *obrms* package is a tool from OpenBabel that calculates RMSD through solving the graph isomorphism problem using a similar algorithm relative to DockRMSD. The values calculated between *obrms* and DockRMSD (if the bond type information is not used in DockRMSD) are identical; therefore, the most poignant comparison between these two programs is to determine how quickly they respectively come to the correct answer. The results of this experiment are summarized in Fig. 5, with runtimes being log-transformed to more closely resemble normal distributions. As is shown, every calculation performed by DockRMSD was faster than the fastest calculation made by *obrms*, which is consistent with the statistically significant difference between their average runtimes (t = 310.6, p < 10^−20,000^ by one-tailed paired t-test). The difference between symmetry-corrected and symmetry-uncorrected runtime is also statistically significant (t = 43.9, p < 10^−400^ by one-tailed paired t-test), but the magnitude of mean difference between DockRMSD and *obrms* (1.04 log_10_(seconds)) is much larger than between symmetry-corrected and naïve runtime (0.21 log_10_(seconds)). This data suggests that while the impact of symmetry correction on RMSD calculation time is observable, its impact on runtime relative to *obrms*, which performs a similar symmetry correction, is minimized.

While a good portion of this runtime difference can be attributed to the fact that *obrms* is implemented using OpenBabel’s object-oriented framework and thus leads to the instantiation of more computationally intensive data structures than is necessary for this problem, DEE also contributes to the increased efficiency of DockRMSD. As an illustrative example of DEE’s power, a buckminsterfullerene (C_60_) molecule was docked onto tRNA-Guanine Transglycosylase [26] using AutoDock Vina, and subsequently, docking RMSD was calculated between the top five poses using DockRMSD without DEE, DockRMSD with DEE, and *obrms* for runtime analysis. The choice of receptor was random and arbitrary in this experiment; docking on this receptor was only a means to generate hypothetical poses for the ligand and implies no greater biological relevance. However, the reason buckminsterfullerene molecule was chosen as the ligand is that it is one of the most highly symmetric molecules that has been observed in nature: each carbon is chemically identical to every other carbon in the molecule, leading to a total state space of 60^60^ possible mappings, a greater number of mappings than there are atoms in the universe. Therefore, proper pruning of the mapping search space is essential to efficiently find the minimum RMSD for this molecule. Reflective of this, DockRMSD without DEE requires a relatively high amount of time (on average 93.3 ± 0.9 ms per ligand pair) to find the optimal solution, as the only pruning done is the bond-based and duplicate criteria described in the implementation; the atom identity search provides no information due to the symmetry of buckminsterfullerene. The *obrms* tool prunes more efficiently (on average 59.6 ± 0.9 ms per ligand pair) due to its direct implementation of the VF2 feasibility criteria, but still needs to enumerate through every valid mapping to find the optimal one and thus takes longer to arrive at the optimal answer. However, since DEE prunes mappings based on their cumulative square distance, DockRMSD is able to find the optimal solution within a timeframe that rivals the runtime of *obrms* on most other molecules (on average 8.7 ± 0.7 ms per ligand pair).

## Conclusion

The inability of naïve RMSD calculation to account for molecular symmetry negatively impacts how we evaluate ligand poses generated by protein–ligand docking. In the dataset we analyzed, about two out of every five ligands require some sort of symmetry correction to achieve accurate docking RMSD values, some of which demonstrated an RMSD correction of more than 2.0 Å. While several attempts have been made to address this need, implementations that find mappings without considering atomic connectivity, such as those in DOCK6 and AutoDock Vina, ultimately fail to consider properties of the ligand that are necessary to find the true optimal symmetry-corrected RMSD. While modules from commercial programs like GOLD and Glide are also capable of finding the optimal solution and are likely more convenient if the poses being evaluated were generated from these programs, users who wish to use these programs must purchase a license or install hefty software packages to perform RMSD calculations. Finally, even when compared to analogous open-source modules, such as *obrms*, DockRMSD has demonstrated much faster calculations in all cases (particularly high-symmetry cases) due to its lightweight implementation. In addition to symmetry correction, the atomic correspondence search of DockRMSD promotes easier comparison between docking programs in benchmarking studies. Ligand poses generated by several programs do not necessarily have direct atomic correspondence, and so DockRMSD could be used as a universal analysis module to ensure all programs are able to be compared and that the comparison is fair.

Despite the ability of DockRMSD to calculate symmetry-corrected RMSD, a few shortcomings of the program remain. For example, DockRMSD requires that the two molecules that are provided are the same molecule due to the atom identity search step. This could potentially be solved through implementation of maximum common substructure searching. However, if the molecule being analyzed is symmetric, a common substructure could potentially correspond to several positions in the molecule, leading to several different potential RMSD values. In addition, DockRMSD currently only evaluates ligand pose distance through docking RMSD because of the popularity of this metric. However, RMSD is far from a perfect metric, particularly because of its inability to capture the conservation of essential protein–ligand interactions that confer high binding affinity. As of now, DockRMSD does not include metrics that address these shortcomings of RMSD because they require the consideration of the protein receptor structure, but future iterations of this software could feasibly incorporate this information along with the typical RMSD calculation.

## Data Availability

The datasets generated and analysed during the current study as well as DockRMSD source code are available at the DockRMSD webserver, https://zhanglab.ccmb.med.umich.edu/DockRMSD/.

## References

[1] Tuccinardi T (2009). Docking-based virtual screening: recent developments. Comb Chem High Throughput Screen.

[2] Śledź P, Caflisch A (2018). Protein structure-based drug design: from docking to molecular dynamics. Curr Opin Struct Biol.

[3] Blum A, Böttcher J, Heine A (2008). Structure-guided design of C2-symmetric HIV-1 protease inhibitors based on a pyrrolidine scaffold. J Med Chem.

[4] Lindberg J, Pyring D, Löwgren S (2004). Symmetric fluoro-substituted diol-based HIV protease inhibitors: ortho-fluorinated and meta-fluorinated P1/P1′-benzyloxy side groups significantly improve the antiviral activity and preserve binding efficacy. Eur J Biochem.

[5] Benach J, Swaminathan SS, Tamayo R (2007). The structural basis of cyclic diguanylate signal transduction by PilZ domains. EMBO J.

[6] Trott O, Olson AJ (2010). AutoDock Vina: improving the speed and accuracy of docking with a new scoring function, efficient optimization, and multithreading. J Comput Chem.

[7] Allen WJ, Rizzo RC (2014). Implementation of the Hungarian algorithm to account for ligand symmetry and similarity in structure-based design. J Chem Inf Model.

[8] Allen WJ, Balius TE, Mukherjee S (2015). DOCK 6: impact of new features and current docking performance. J Comput Chem.

[9] Kuhn HW (1955). The Hungarian method for the assignment problem. Nav Res Logist Q.

[10] Munkres J (1957). Algorithms for the assignment and transportation problems. J Soc Ind Appl Math.

[11] Jones G, Willett P, Glen RC (1997). Development and validation of a genetic algorithm for flexible docking. J Mol Biol.

[12] D.A. Case, I.Y. Ben-Shalom, S.R. Brozell, D.S. Cerutti, T.E. Cheatham, III, V.W.D. Cruzeiro TAD, R.E. Duke, D. Ghoreishi, M.K. Gilson, H. Gohlke, A.W. Goetz, D. Greene, R Harris, N. Homeyer YH, S. Izadi, A. Kovalenko, T. Kurtzman, T.S. Lee, S. LeGrand, P. Li, C. Lin, J. Liu, T. Luchko, R. Luo DJ, et al. AMBER 2018; 2018

[13] Friesner RA, Banks JL, Murphy RB (2004). Glide: a new approach for rapid, accurate docking and scoring. 1. Method and assessment of docking accuracy. J Med Chem.

[14] Halgren TA, Murphy RB, Friesner RA (2004). Glide: a new approach for rapid, accurate docking and scoring. 2. Enrichment factors in database screening. J Med Chem.

[15] O’Boyle NM, Banck M, James CA (2011). Open Babel: an open chemical toolbox. J Cheminform.

[16] Vento M, Cordella LP, Foggia P, Sansone C (2004). A (sub) graph isomorphism algorithm for matching large graphs. IEEE Trans Pattern Anal Mach Intell.

[17] Hu J, Liu Z, Yu DJ, Zhang Y (2018). LS-align: an atom-level, flexible ligand structural alignment algorithm for high-throughput virtual screening. Bioinformatics.

[18] RDKit: Open-source cheminformatics. http://www.rdkit.org

[19] DockRMSD: docking pose distance calculation. https://zhanglab.ccmb.med.umich.edu/DockRMSD/

[20] Desmet J, De Maeyer M, Hazes B, Lasters I (1992). The dead-end elimination theorem and its use in protein side-chain positioning. Nature.

[21] Dunbar JB, Smith RD, Yang CY (2011). CSAR benchmark exercise of 2010: selection of the protein-ligand complexes. J Chem Inf Model.

[22] Pettersen EF, Goddard TD, Huang CC (2004). UCSF Chimera—a visualization system for exploratory research and analysis. J Comput Chem.

[23] Wong DM, Greenblatt HM, Dvir H (2003). Acetylcholinesterase complexed with bivalent ligands related to Huperzine A: experimental evidence for species-dependent protein–ligand complementarity. J Am Chem Soc.

[24] munkres - Munkres implementation for Python. http://software.clapper.org/munkres/index.html

[25] Andersson HO, Fridborg K, Löwgren S (2003). Optimization of P1–P3 groups in symmetric and asymmetric HIV-1 protease inhibitors. Eur J Biochem.

[26] Biela I, Tidten-Luksch N, Immekus F (2013). Investigation of specificity determinants in bacterial tRNA-guanine transglycosylase reveals queuine, the substrate of its eucaryotic counterpart, as inhibitor. PLoS ONE.

